# Complete mitochondrial genome of *Coleophora therinella* Tengström, 1848 (Lepidoptera: Coleophoridae)

**DOI:** 10.1080/23802359.2021.1944387

**Published:** 2021-07-02

**Authors:** Jeong Sun Park, Min Jee Kim, Sung- Soo Kim, Iksoo Kim

**Affiliations:** a Department of Applied Biology, College of Agriculture and Life Sciences, Chonnam National University, Gwangju, Republic of Korea; b Experiment and Analysis Division, Honam Regional Office, Animal and Plant Quarantine Agency, Gunsan, Republic of Korea; c Research Institute for East Asian Environment and Biology, Seoul, Republic of Korea

**Keywords:** Mitochondrial genome, Coleophoridae, *Coleophora therinella*, Gelechioidea

## Abstract

The mitochondrial genome (mitogenome) of *Coleophora therinella* Tengström, 1848 is the first report for the family Coleophoridae in Lepidoptera. The 15,539-bp long complete genome has an arrangement identical to that observed in most lepidopteran genomes. *COI* had the atypical CGA codon that is frequently found in the start region of the lepidopteran *COI,* and *COII* had the GTG codon found previously in *Drosophila yakuba ND5* and *Rattus norvegicus ND1*. The 457-bp long A + T-rich region was the second largest, next to *Blastobasis lacticolella*, which belongs to Blastobasidae in the superfamily Gelechioidea. The A/T content of the whole mitogenome was 80.7%; however, it varied among the regions/genes as follows: A + T-rich region, 94.8%; *srRNA*, 85.0%; *lrRNA*, 84.3%; tRNAs, 81.5%; and PCGs, 78.9%. Phylogenetic analyses with concatenated sequences of the 13 PCGs and two RNA genes using the maximum likelihood method, placed Coleophoridae, represented only by *C. therinella*, as the most basal lineage of the Gelechioidea families consisted of Stathmopodidae, Scythrididae, Blastobasidae, Autostichidae, and Oecophoridae, but nodal support for this grouping was very low (27%). Currently, several families of Gelechioidea are represented by a single species. Thus, extended sampling is required for further reasonable inference for the relationships of these families.

*Coleophora therinella* Tengström, 1848 belongs to Coleophoridae in Lepidoptera. The genus *Coleophora*, which is composed of 400–500 species is commonly known as ‘casebearers’ because the larvae of most species construct distinctive portable protective silken cases (Coshan 2009). The species is found in Central Asia including Korea, Europe, and Siberia (Park and Baldizzone 1992; Anikin 1998; Baldizzone et al. 2006; Kim et al. 2013) (Figure 1).

**Figure 1.**
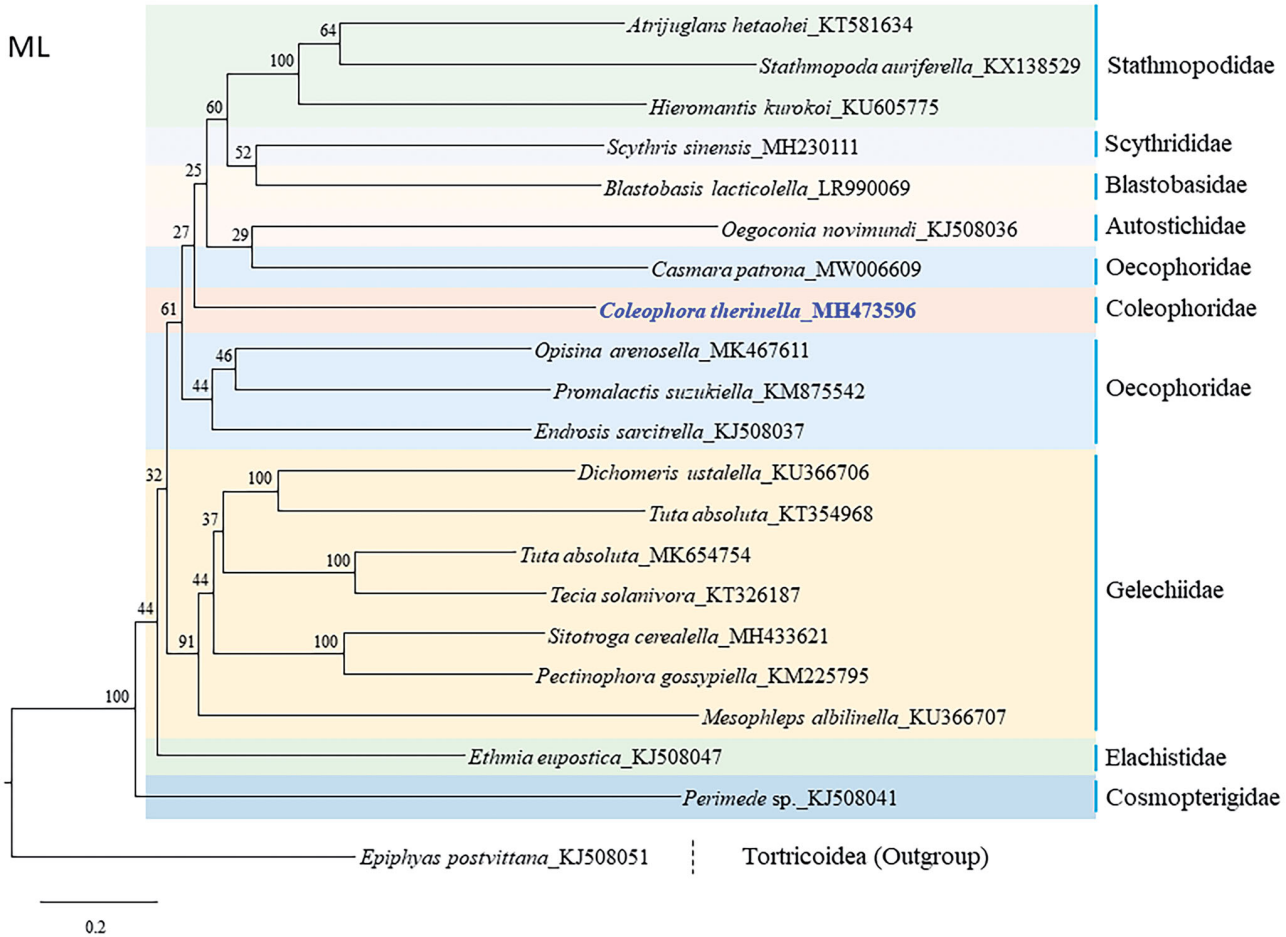
Phylogenetic tree for Gelechioidea. The tree was constructed using nucleotide sequences of 13 protein-coding genes and two rRNAs via the maximum likelihood method. The numbers at each node specify bootstrap percentages of 1000 pseudoreplicates. The scale bar indicates the number of substitutions per site. Tortricoidea (*Epiphyas postvittana*, KJ508051, Timmermans et al. 2014) was used as the outgroup. GenBank accession numbers are as follows: *Mesophleps albilinella*, KU366707 (Park et al. 2016b); *Dichomeris ustalella*, KU366706 (Park et al. 2016b); *Pectinophora gossypiella*, KM225795 (Zhao et al. 2016); *Sitotroga cerealella*, MH433621 (Yuan et al. 2019); *Tecia solanivora*, KT326187 (Ramírez-Ríos et al. 2016); *Helcystogramma macroscopa*, KT354968 (Ma et al. 2016); *Tuta absoluta*, MK654754 (Zhang et al. 2019); *Ethmia eupostica*, KJ508047 (Timmermans et al. 2014); *Perimede* sp., KJ508041 (Timmermans et al. 2014); *Endrosis sarcitrella*, KJ508037 (Timmermans et al. 2014); *Promalactis suzukiella*, KM875542 (Park et al. 2016c); *Opisina arenosella*, MK467611 (Meng et al. 2019); *Casmara patrona*, MW006609 (Unpublished); *Oegoconia novimundi*, KJ508036 (Timmermans et al. 2014); *Blastobasis lacticolella*, LR990069 (Unpublished); *Hieromantis kurokoi*, KU605775 (Park et al. 2016a); *Stathmopoda auriferella*, KX138529 (Jeong et al. 2016); *Atrijuglans hetaohei*, KT581634 (Wang et al. 2016); *Scythris sinensis*, MH230111 (Park et al. 2020).

An adult male *C. therinella* was collected from Geoje City, Gyeongsangnam-do Province (34°48'29.2″N, 128°38'2.5″E), South Korea in 2012. This voucher specimen and DNA were deposited at the Chonnam National University, Gwangju, Korea, under the accession no. CNU6202 (Iksoo Kim, ikkim81@chonnam.ac.kr). Using DNA extracted from the hind legs, three long overlapping fragments (LFs; *COI*-*ND4*, *ND5*-*lrRNA*, and *lrRNA*-*COI*) were amplified and used as templates for the amplification of 26 short overlapping fragments using the primers reported in Kim et al. (2012).

Phylogenetic analysis was performed using the concatenated nucleotide sequences of 13 protein-coding genes (PCGs) and two rRNA genes of 20 mitogenome sequences available from Gelechioidea in Lepidoptera, including that of *C. therinella*. The maximum likelihood (ML) method that is implemented in CIPRES Portal v. 3.1 (Miller et al. 2010) was used for the phylogenetic analysis. An optimal partitioning scheme (6 partitions) and substitution model (GTR + Gamma + I) were determined using PartitionFinder 2 and the Greedy algorithm (Lanfear et al. 2012, 2014, 2016).

The complete 15,539-bp mitogenome of *C. therinella* was composed of typical sets of genes (two rRNAs, 22 tRNAs, and 13 PCGs) and a major non-coding 457 bp A + T-rich region (GenBank acc. no. MH473596), with the gene arrangement being identical to that observed in most lepidopteran genomes (Kim et al. 2010). The length of the *C. therinella* A + T-rich region was the second largest among sequenced Gelechioidea, which ranged in other species of Gelechioidea from 271 (*Scythris sinensis* in Scythrididae; Park et al. 2020) to 626 bp (*Blastobasis lacticolella* in Blastobasidae; unpublished, GenBank acc. no. LR990069). Eleven PCGs had the typical ATN start codon, whereas *COI* had an atypical CGA codon that is frequently found in the start region of the lepidopteran *COI,* and *COII* had the GTG codon found previously in *Drosophila yakuba ND5* and *Rattus norvegicus ND1* (Clary and Wolstenholme 1985; Gadaleta et al. 1988). The A/T content of the whole mitogenome was 80.57%, well within the range found in Gelechioidea (77.6% in Park et al. 2016a,b,c and 81.5% in Timmermans et al. 2014), and varied among the region/genes as follows: the A + T-rich region, 94.8%; *srRNA*, 85.0%; *lrRNA*, 84.3%; tRNAs, 81.5%; and PCGs, 78.9%.

Phylogenetic analysis placed Coleophoridae, represented by the current *C. therinella* to a group, consisted of Stathmopodidae, Scythrididae, Blastobasidae, Autostichidae, and Oecophoridae, but nodal support for this grouping was very low (27%). Strong support was obtained only for the monophylies of Stathmopodidae (100%) and Gelechiidae (91%), respectively. Currently, only 20 species belonging to nine families are available for their mitogenome sequences in Gelechioidea, including that of *C. therinella*. Thus, most families are represented by one-three mitogenome sequences, excluding the family Gelechiidae. Therefore, an inference of phylogenetic relationships among the families of Gelechioidea is unavoidably limiting. Thus, more mitogenome sequences from a diverse taxonomic group are required for further reasonable inference for the relationships of the families in Gelechioidea.

## Data Availability

The genome sequence data that support the findings of this study are openly available in GenBank of NCBI at https://www.ncbi.nlm.nih.gov/nuccore/MH473596.1
